# Annotations

**Published:** 1894-04-14

**Authors:** 


### Annotations.

### The Scientific Method.

Professor Ira Remsen speaking at the opening of the Kent Chemical Laboratory of the University of Chicago a few weeks ago, said,—"It is curious that the scientific method of work, which is altogether the simplest, should be the last to be adopted by the world as it is by individuals. . . . One of the immediate causes is undoubtedly the fact that at an early period in history those who worked with their hands came to be looked upon as inferior to those who worked with their heads alone." Put this by the side of the late Sir Andrew Clark's utterance, that whilst he sometimes admired our medical workers in physiological and pathological laboratories, he distrustedtheir results, because he felt that as a rule they knew nothing at all of the actual work of the bedside. The plain truth is that the physiological laboratory man thinks his work infinitely superior to that of the bedside man, and actually fears the loss of caste if he should show any knowledge of clinical work. Of course, than this, nothing more absurd was ever dreamt of, even in a lunatic asylum. What is really wanted for the due fruitfulness of the physiological and pathological laboratory is a wide and deep experimental knowledge of bedside work. One cannot help feeling that the experimental physiologist would be exceedingly advantaged by at least half a dozen years of personal clinical experience after the obtaining of his degree. Half of the six years should be spent in the general and special wards of a hospital, and the other half in the general practice of medicine in town or country. We commend this suggestion to the licensing department and all teaching clinicians and experienced practitioners who deplore the futility of laboratory work and the decay of "the scientific method" in the art and practice of common medicine.

### The Chelsea Hospital and Dr. Parkes.

The case of the Chelsea Hospital for Women and Dr. Parkes' contention therewith has been studied by us with minute care, and we can come to no other conclusion than that with regard to the medical staff of the hospital Dr. Parkes has behaved in a manner opposed to all the canons of professional brotherliness, and with regard to the lay authorities his conduct has set at nought every axiom of common sense. The business at the outset seems to have been as simple as a rule of three sum. There were three cases of scarlet fever at the hospital, there was some septicæmia, there was excessive mortality after operations. Clearly something was wrong. But all that was wrong could have been set right easily and promptly by the loyal co-operation of the hospital authorities with the medical officer of health, Dr. Parkes. That co-operation seemed to be in full activity, and all was tending towards the natural and unexciting end of an unfortunate incident, when Dr. Parkes suddenly, and, so far as we can see, without any justification whatever, kicked down the scaffolding of peace and threw the whole structure of progressive improvement to the ground. He committed the very grave error of dragging his professional brethren of the hospital staff before an incompetent lay tribunal, the Chelsea Vestry, and made the unheard of and preposterous demand that the surgical operations of a hospital should be placed under the control of the medical officer of health for the district in which the hospital happened to be placed. Nothing more need be said. To state the case is to condemn the conduct of Dr. Parkes. There is only one manly and straightforward course to be taken. Dr. Parkes has committed a grave error of judgment. He should immediately withdraw from his untenable position, and if he resolves, as he should, to withdraw like a gentleman, he will make an ample apology both to the medical staff and to the lay managers of the hospital.

### Should Journalists Marry?

Two or three weeks ago the question, from a physiological standpoint, was propounded in The Hospital—should journalists marry? and it was attempted to be shown that there were at least some reasons why they should not. The *Journalist* itself and other newspapers have dealt with our brief article, and not entirely in terms of approval. We venture, however, to ask for the second thoughts of the newspaper press. To begin with, it can hardly be doubted that newspaper writers exercise more influence than any other class of persons in the formation of men's opinions. Civilisation, that is, scientific civilisation, with Britain in the front rank, is, we hold, quite in its infancy. Its head nurse is the journalist. Our point is that it would be well for this infant civilisation if its head nurse could devote itself entirely and absolutely to the well-being of its charge; if it could be so circumstanced as to have no private interests or anxieties to impair capacity or lessen devotedness. Of course, we are aware that this is a counsel of perfection, and, perhaps, we should have suggested, not that journalists as a class should refrain from marriage, but that a picked hundred or so of the most heroic souls in British journalism should, so to speak, live, and if need be, die, as soldiers do every day, for their nation and civilisation. We are, we repeat, at a crude period. The masses of the people have only just begun to live. They need elementary, but high and noble guidance in every department of public and private activity; in patriotism, in political economy, in common justice between man and man, class and class, in a rational philosophy of life, in detailed private morals, in a reasonable religion. These are tasks worthy of any man's ambition, and of any sacrifice, the very greatest, which their successful carrying out may demand. Is it, or is it not the case, that in almost every department of journalism the journalist must please, first his editor and then his readers? If his prime aim is to please, it cannot also be to enlighten, to convince, to persuade, to compel to that which is truest and highest in thought and action. When a man has only himself to think of he can sacrifice himself in the cause of country and civilisation, without too much effort. But when he has children and a wife, the task, which for himself would be easy, becomes all but impossible. We cannot but feel that journalism, as much as arms, science, or religion, may demand its self-sacrificing heroes; and we are quite convinced that when the necessity arises, those heroes, at any rate in England, will not be found skulking in the rear.

